# From the integrated stress response to oxidative stress: A historical perspective

**DOI:** 10.1016/j.jbc.2025.110958

**Published:** 2025-11-19

**Authors:** Mehdi Amiri, Phoenix Toboz, Michael A. Bellucci, Soroush Tahmasebi, Nahum Sonenberg

**Affiliations:** 1 Rosalind and Morris Goodman Cancer Institute, McGill University, Montreal, Quebec, Canada; 2 Department of Biochemistry, Faculty of Medicine and Health Sciences, McGill University, Montreal, Quebec, Canada; 3 Department of Pharmacology and Regenerative Medicine, University of Illinois at Chicago, Chicago, Illinois, USA; 4 University of Illinois Cancer Center, University of Illinois, Chicago, Illinois, USA

**Keywords:** eIF2, glutathione, ISR, mRNA translation, oxidative stress

## Abstract

Oxidative stress has exerted fundamental evolutionary pressure since the emergence of aerobic life. Its impact on the physiology and function of all organisms is profound and consequential for cell survival. The integrated stress response (ISR) plays a critical role in counteracting oxidative stress *via* translational control of a subset of mRNAs. Here, we summarize the fundamental discoveries that shaped our understanding of the ISR pathway's role in cellular adaptation to oxidative stress from studies of protein synthesis in reticulocyte lysates to the regulation of glutathione metabolism downstream of the ISR pathway. We describe recent advances in studying mRNA translation changes in response to oxidative stress based on high throughput translatome analyses.

Since the evolution of aerobic life, cells have perenially faced oxidative stress. Reactive oxygen species (ROS), generated either in response to extracellular stressors (*e.g.*, toxins, radiation) or as byproducts of intracellular processes such as mitochondrial respiration and metabolism, lead to cellular damage and age-related disorders (1). In response to oxidative stress, multiple signaling pathways cooperate to protect cells from the detrimental effects of ROS. In mammals, one of the central protective mechanisms involves glutathione (GSH), the most abundant antioxidant in mammalian cells (2). The complex interplay between the integrated stress response (ISR) pathway and GSH metabolism is instrumental in facilitating organisms to adapt to oxidative insults.

The ISR is a highly conserved mechanism that protects cells and organisms from both external and internal stressors (Fig. 1*A*). A cardinal component of this pathway is the eukaryotic translation initiation factor 2 (eIF2). eIF2 forms a ternary complex (TC) with GTP and Met-tRNAi and is recruited to the small ribosomal subunit during translation initiation to locate the start codon (3). Upon engagement with the translation initiation codon, GTP is hydrolyzed to GDP. The resulting eIF2•GDP is more stable than eIF2•GTP, and thus, the regeneration of eIF2•GTP requires the action of a guanine nucleotide exchange factor (GEF) known as eIF2B to reconstitute the TC (4, 5, 6). Under cellular stress conditions, the α subunit of eIF2 (eIF2α), encoded by the *EIF2S1* gene, is phosphorylated at a conserved serine residue (serine 52, historically mischaracterized as serine 51 (7, 8)) by eIF2α kinases (eIF2AKs). As a result, eIF2α is converted from a substrate to an inhibitor of eIF2B, thereby halting the initiation of mRNA translation (9, 10). Translation inhibition is not global, as paradoxically, a subset of mRNAs is preferentially translated under stress conditions (11, 12). In mammals, activating transcription factor 4 (ATF4) is the most well-studied factor upregulated in response to eIF2α phosphorylation under cellular stress (13, 14, 15, 16).

**Figure 1:**
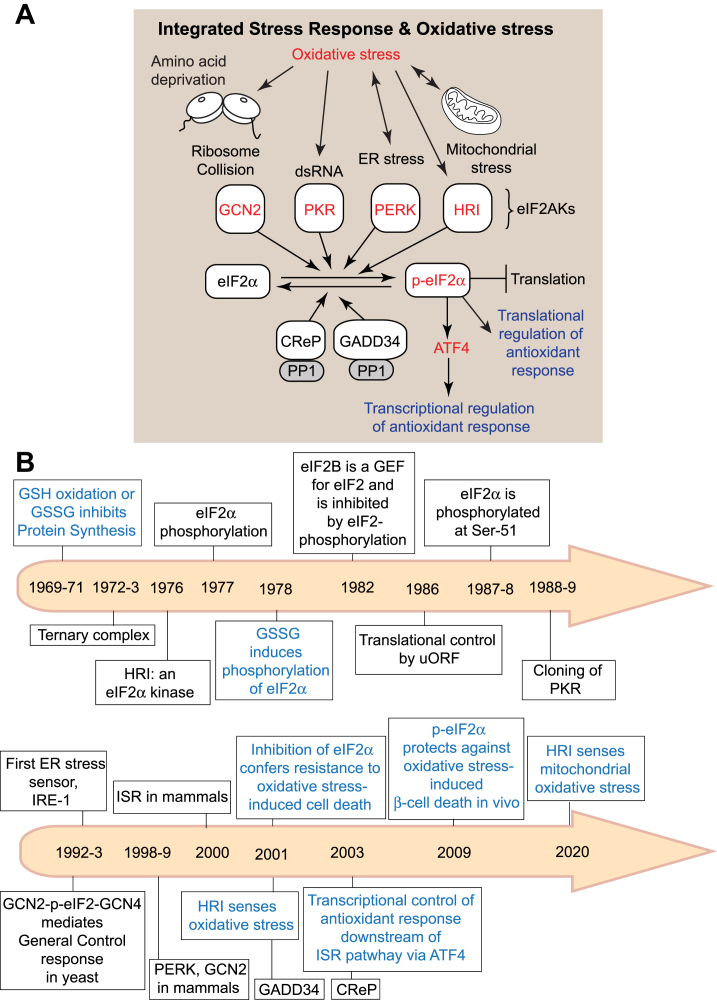
**The integrated stress response and oxidative stress.***A*, activation of eIF2AKs (HRI, PKR, PERK, or GCN2) by oxidative stress induces phosphorylation of the α subunit of eIF2. eIF2α phosphorylation is reversible through the activity of two phosphatases, GADD34:PP1 and CReP: PP1. eIF2α phosphorylation inhibits global translation, but the translation of a subset of mRNAs, such as ATF4 and mRNAs involved in antioxidant response, increases. ATF4 promotes expression of the genes involved in GSH metabolism and antioxidant response. *B*, timeline of discoveries in the fields of the ISR and oxidative stress. Major advances are shown according to the year of publication.

This pathway is referred to as the ISR to highlight the convergence of diverse cellular stress into a common response which is the phosphorylation of eIF2α (14, 17) (Fig. 1*A*). Mammalian cells express four eIF2α kinases (eIF2AK1–4), each of which responds to distinct stress signals (17). eIF2AK1 (heme-regulated inhibitor, or HRI) responds to heme deficiency, oxidative stress, and mitochondrial stress; eIF2AK2 (double-stranded RNA-activated protein kinase, or PKR) is activated by double-stranded RNA (dsRNA); eIF2AK3 (PKR-like endoplasmic reticulum kinase, or PERK) senses unfolded proteins in the endoplasmic reticulum (ER); and eIF2AK4 (general control nonderepressible 2, or GCN2) is activated by uncharged tRNAs, UV radiation, and ribosome collision (17, 18, 19). The path leading to the discovery of the ISR was closely intertwined with studies of oxidative stress and GSH metabolism (Fig. 1*B*). Here, we provide a historical perspective on the key discoveries and recent findings that advanced the field.

## Milestone discoveries linking oxidative stress to the ISR

### Early years of translation control, oxidative stress, and the ISR

**Figure 2:**
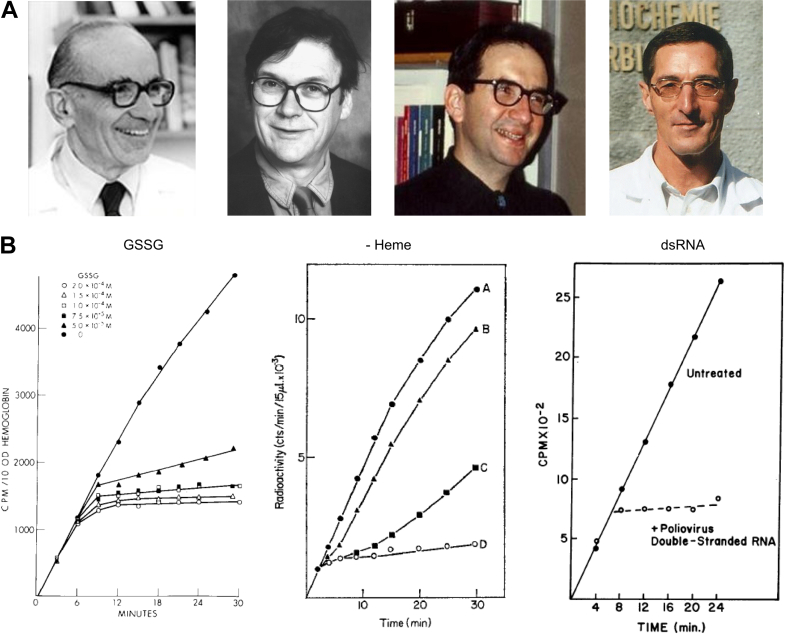
**Early evidence for inhibition of translation initiation by oxidative stress.***A*, photographs (from left) of Irving M. London, Tim Hunt, Edward M. Kosower, and Hans Trachsel. *B*, GSSG inhibits protein synthesis similar to dsRNA and a lack of heme. *Panels* were adopted from (25, 97, 98). Heme (middle) panel hemin-deficient mixtures were made, and hemin was added to the the mixture at zero time (A), after 1 min (B), and after 5 min (C). D represents the control (no hemin added).

Early evidence linking oxidative stress and GSH metabolism to eIF2-mediated translational control emerged over 50 years ago through studies of globin synthesis in a cell-free translation system (20). In 1968, Tim Hunt (the co-awardee of the 2001 Nobel prize in Physiology or Medicine) joined Irving M. London’s lab to study heme-mediated regulation of protein synthesis using a rabbit reticulocyte lysate (Fig. 2*A*). At that time, it was already known that removing heme from reticulocyte lysate inhibits globin protein synthesis (21). The development of a new class of thiol-oxidizing agents revealed that protein synthesis is temporarily suppressed in mammalian cells when approximately 80% of GSH is oxidized (22, 23). Hunt, in collaboration with Nechama and Edward Kosower, made the seminal discovery that oxidized glutathione (GSSG) inhibits protein synthesis, similar to thiol-oxidizing agents such as diamide (24, 25). Strikingly, the inhibition kinetics closely mirrored those observed following heme deficiency or exposure to dsRNA (Fig. 2*B*) (25, 26, 27). This intriguing resemblance suggested that all three agents inhibit protein synthesis through a common mechanism. Shortly thereafter, eIF2 was purified in complex with Met-tRNA_i_ and the 40S ribosomal subunit (28), and the first eIF2 kinase (HRI, eIF2AK1) was purified and characterized independently by Irving London’s and Boyd Hardesty’s labs (29, 30). These findings established the paradigm that eIF2 phosphorylation, which was identified by Trachsel’s lab (Fig. 2*A*), is the common mechanism by which heme deficiency, dsRNA, and GSSG inhibit protein synthesis (31, 32). While the mammalian cell-free translation system was momentous for discovering translation initiation factors and upstream regulators of the eIF2 pathway (33), its lack of a nucleus limited insights into the downstream consequences of eIF2 activation. Breakthrough advances on the latter were made using yeast genetics (see below).

### The protective effect of translation inhibition by eIF2α phosphorylation against oxidative stress

Translational control plays a crucial role in neuronal damage and cell death caused by oxidative stress. The first evidence of this was provided in rats, as cycloheximide, a translation elongation inhibitor, dramatically reduced neuronal damage caused by transient ischemia in the forebrain (34). How could inhibition of mRNA translation benefit cell survival? Soon after, several biochemical and genetic studies provided evidence supporting the importance of inhibiting mRNA translation—either through eIF2α phosphorylation or regulation of eIF2α availability—in protecting mammalian cells and organisms against oxidative stress (35, 36). One surprising piece of evidence came from studies of the hibernating ground squirrel *Spermophilus tridecemlineatus* (35). During hibernation, brain blood flow in squirrels drops to roughly 10% of baseline levels without causing any neurological defects (35). John Hallenbeck and colleagues, using the hibernation cycle of ground squirrels as a natural model of ischemic tolerance, discovered that the rate of protein synthesis in the brains of hibernating squirrels is significantly reduced (35). They also showed that the diminished mRNA translation coincided with an increase in eIF2α phosphorylation, providing strong evidence for the protective role of eIF2α phosphorylation in animal survival during oxidative stress. Using a genetic screen to identify genes that confer resistance to oxidative stress-induced programmed cell death, Schubert and Maher demonstrated that cells expressing low levels of eIF2α are resistant to cell death (36). Through a series of elegant experiments, they demonstrated that eIF2α phosphorylation, like the downregulation of eIF2α, protects neuronal cells against oxidative stress by increasing GSH levels. Mechanistically, they demonstrated that phosphorylation of eIF2α promotes the translation of γ-glutamylcysteine synthetase, a rate-limiting enzyme in GSH biosynthesis (36).

### Discovery of the ISR pathway and transcriptional regulation of antioxidant response *via* ATF4

During the last decade of the 20th century, two lines of research emerged: one focusing on ER chaperones in mammalian cells and the other on starvation responses in yeast. By the year 2000, investigators from these seemingly separate fields pieced together the signaling pathway now recognized as the ISR (12, 13, 14, 37, 38, 39, 40). Unlike the cytosol, the ER maintains a highly oxidative environment. The ratio of GSH to GSSG is approximately 1:1 to 3:1 in the ER, compared to 30:1 to 100:1 in the cytosol (41). While this unique compartmentalization facilitates proper protein folding and disulfide bond formation in the ER, it also generates a significant portion of cellular ROS—approximately 25% —which consumes the cellular GSH pool (42). Changes in the redox status or protein-folding environment of the ER trigger ER stress (43).

**Figure 3:**
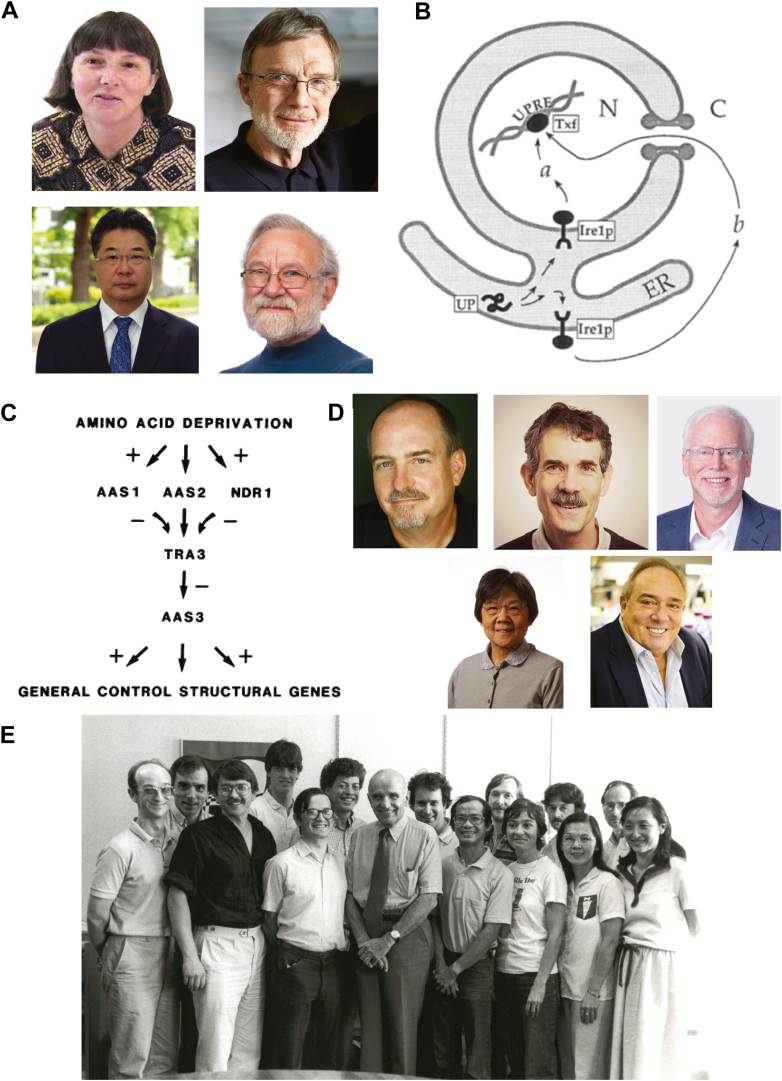
**Discovery of the UPR and the ISR.***A*, Photographs of Mary-Jane Gething (*upper left*), Joe Sambrook (*upper right*), Kazutoshi Mori (*lower left*), and Peter Walter (*lower right*). *B*, an early model of the UPR proposed by Peter Walter in 1993 (38). *C*, an early model of the ISR pathway proposed by Hinnebusch in 1983 (48) by studying mutations that confer sensitivity or resistance to starvation by decreasing or increasing, respectively, the translation of *AAS3* mRNA and attendant transcriptional activation by the *AAS3* product in starved cells of *Saccharomyces cerevisiae*. *AAS1*, *AAS3*, and *NDR1* were renamed as *GCN2*, *GCN4*, and *GCN1*, respectively. *AAS2* and *TRA3* were renamed as *GCN3* and *GCD1*, which are the alpha and gamma subunits of eIF2B, respectively. *D*, *Top*: Photographs of Alan Hinnebusch (*left*), David Ron (*center*), and Ronald Wek (*right*). *Bottom*: Photographs of Jane-Jane Chen (*left*) and Randal J. Kaufman (*right*). *E*, the London lab group meeting taken in the summer of 1983. Six people who were involved in studying ISR were Irving M London (fourth from *left*, front row), Jane-Jane Chen (second from *right*, front row), Daniel H Levin (first from *right*, back row), Robert L Matts (third from *right*, back row), Ray Petryshyn (second from *left*, front row), N. Shaun B Thomas (first from *left*, front row).

By the turn of the 21st century, several groundbreaking discoveries established the link between ER stress and the transcriptional control of the antioxidant response. Although it had been known for several decades that various cellular stresses (*e.g.*, Rous sarcoma virus transformation and glucose deprivation) promote the expression of a subset of ER proteins, the significance of these findings remained unclear (44, 45). In 1988, Mary-Jane Gething and Joseph Sambrook discovered that the accumulation of misfolded proteins in the ER elicits a transcriptional response, which serves as the primary trigger for the expression of BiP/GRP78—the most abundant protein chaperone in the ER (40) (Fig. 3*A*). Subsequent work by Kazutoshi Mori, a postdoctoral fellow in Gething and Sambrook’s labs, and others, led to the discovery of the unfolded protein response (UPR) pathway. One of the key findings related to the UPR was the identification of IRE1 (inositol-requiring enzyme 1), the first ER stress sensor, by Peter Walter and Kazuhiro Mori (38, 39) (Fig. 3*B*). The search for a protein with IRE1-like luminal domain and PKR/HRI-like kinase domain led to the discovery of PERK by the Ron lab (46). PERK was found to be identical to a pancreatic-enriched eIF2α kinase identified by the Shi and Wek labs (47), though its role in the response to ER stress was not appreciated at the time.

In parallel with discoveries related to the unfolded protein response (UPR), Alan Hinnebusch and others uncovered translational control mechanisms downstream of eIF2α phosphorylation. At that time, the General Control response in the yeast *Saccharomyces cerevisiae* was known as a common regulatory mechanism that promotes the expression of many genes involved in amino acid metabolism in response to starvation. Studying mutations in *GCN* (General Control Nonderepressible) that interfered with yeast’s ability to respond to starvation, Thomas E Dever and Hinnebusch noted the similarity between the catalytic domain of yeast GCN2 and the mammalian kinases HRI and PKR and showed that mammalian eIF2 kinases could functionally substitute for yeast GCN2 (37). Hinnebusch further discovered that GCN2-dependent phosphorylation of eIF2α promotes the translation of the mRNA encoding the stress response transcription factor GCN4 (12, 48) (Fig. 3*C*). The identification of the mammalian orthologs of this pathway, including the discovery of ATF4 as the counterpart of GCN4 8 years later by Heather P Harding and David Ron and others, highlighted the striking evolutionary conservation of this ancient pathway (13, 14, 15, 16) (Fig. 3*D*).

The term, Integrated Stress Response, was introduced in Ron’s 2003 paper (14). The work clarified a key feature of animal cell physiology: diverse and seemingly unrelated upstream stress signals converge—via four known eIF2α kinases—on a singular event, eIF2α phosphorylation, which is the mammalian counterpart to the yeast General Control response previously studied by Alan Hinnebusch. Ron’s lab demonstrated that the increase in ATF4 (the mammalian ortholog of GCN4) downstream of the PERK–phospho-eIF2α pathway activates an antioxidant transcriptional program critical for cell survival (14). They showed that cells lacking PERK, in the presence of ER stress, accumulate ROS, an effect revoked by supplementation of cystine and reducing agents such as β-mercaptoethanol and dithiothreitol (DTT). At the turn of the 21st century, several animal models were developed to deplete components of the ISR pathway, including the PKR^−/−^ mouse (49, 50), the HRI^−/−^ mouse (51), the PERK^−/−^ mouse (52), the eIF2α^A/A^ mouse (53), GCN2^−/−^ mouse (54) and the ATF4^−/−^ mouse (55) which provided the tools to examine the interconnections between the ISR and oxidative stress. The Chen lab at MIT, using HRI-null reticulocytes and fetal liver erythroid cells, demonstrated that HRI is the primary eIF2α kinase that senses ROS upon arsenite exposure through a mechanism involving the heat shock protein chaperones Hsp90 and Hsc70 (56). (Fig. 3, *D* and *E*). Further evidence linking eIF2α phosphorylation to antioxidant effects in animals came from studies by Randal J. Kaufman (Fig. 3*D*) in mice expressing a homozygous Ser51Ala mutant of eIF2α specifically in β cells (57). The animals developed severe diabetes due to the loss of β cells. Strikingly, a diet containing antioxidants improved both glucose intolerance and β cell loss (57).

## Fresh concepts in the ISR and oxidative stress

### GCN2 and oxidative stress

Over 50 years ago in Gerald R. Fink’s lab, GCN2 mutation was identified among a handful of mutations that inhibit the derepression of amino acid biosynthetic genes in *S. cerevisiae* (58). Identification of GCN2 sequence by Roussou *et al.* (59), and Wek *et al.* (60) revealed that GCN2 is a protein kinase, and its kinase activity is stimulated by the binding of uncharged tRNA to HisRS-like domain, providing a rationale for how GCN2 could be activated under amino acid starvation conditions. The discovery of mammalian homologue of GCN2 (61, 62) and GCN2^−/−^ mouse (54) paved the way to uncover the importance of this nutrient sensor kinase in oxidative stress.

Diverse stressors, including UV irradiation, amino acid deprivation, and oxidative stress, cause ribosome stalling and collision (63, 64). Studies in the past decade uncovered that ribosome collision triggers the ISR *via* recruitment and activation of GCN2 (63, 64, 65). Ribosome profiling (also known as Ribo-seq) in fission yeast revealed that oxidative stress leads to ribosome stalling on tryptophan codons in response to a decrease in charged tRNA-Trp levels, leading to activation of GCN2 (66). At high levels, nitric oxide (NO), a gaseous, endogenous cellular signaling molecule can impair protein synthesis (67). Reactive nitrogen species (RNS) are formed when NO reacts with other radicals, including ROS. The NO-induced stress causes ribosome collision, which activates ribosomal surveillance pathways including ribotoxic stress response (RSR), ribosome-associated quality control (RQC) and the ISR (67). Ribo-seq and disome-seq in *S. cerevisiae* demonstrated that oxidative stress causes motif-specific (isoleucine-proline sequences) ribosome pausing and leads to ribosome collisions. Rad6, an E2 ubiquitin conjugase, is required for ribosome pausing, translational repression, and promotes phosphorylation of eIF2α in response to oxidative stress (68). Our group has recently uncovered another aspect of the antioxidant effect of the eIF2α pathway mediated by GCN2 (69). We demonstrated that GCN2 knockout mice exposed to hypoxia, amino acid deprivation, or phenylhydrazine (a hemolytic agent with strong oxidative activity) develop defects in red blood cell clearance by liver macrophages. Furthermore, we showed that GCN2-dependent regulation of the antioxidant NRF2-HO1 axis plays a critical role in mediating this effect, supporting previous reports identifying GCN2 as a redox regulator (70).

### Mitochondrial oxidative stress and the ISR

Mitochondria are the major source of ROS (∼90% of cellular ROS) (71), as byproducts of oxidative phosphorylation. Mitochondrial ROS and other mitochondrial stress conditions, such as inhibition of mitochondrial translation by doxycycline (72) or disruption of mitochondrial membrane potential by the ionophore carbonyl cyanide-4-(trifluoromethoxy)phenylhydrazone (FCCP) (73), trigger ATF4 expression in an HRI-dependent manner (74, 75, 76). Notably, activation of HRI in response to mitochondrial stress is independent of cytosolic ROS (77, 78). In search for a signaling pathway that relays mitochondrial stress to the ISR pathway, two groups employed genome-wide screening using CRISPR interference or random mutagenesis by gene trapping (77, 78). They identified the mitochondrial protease OMA1 (overlapping activity with m-AAA protease 1, also known as OMA1 Zinc Metallopeptidase) and the poorly characterized protein DELE1 (also known as DAP3 binding cell death enhancer 1) as specific sensors of mitochondrial stress that activate HRI. Both OMA1 and DELE1 localize to the inner mitochondrial membrane, and in response to mitochondrial stress, OMA1 cleaves DELE1, which then interacts with and activates HRI (77, 78).

The ISR also couples mitochondrial protein synthesis and oxidative stress through PERK-eIF2α activation (79). Mitochondrial ROS, which is generated in cardiomyocytes following ischemia/reperfusion (I/R), causes cardiac reperfusion injury, which is ameliorated by PERK-eIF2α activation. Specifically, PERK-eIF2α activation suppressed translation of mitochondrial complex mRNAs, including NDUFAF2 (NADH:ubiquinone oxidoreductase complex assembly factor 2), a mitochondrial complex I assembly factor. Reduced NDUFAF2 expression *via* PERK signaling resulted in decreased ROS and increased cell survival in response to I/R both *in vitro* and *in vivo* (79). This highlights the importance of the ISR signaling in response to mitochondrial ROS and in maintaining mitochondrial homeostasis.

### Transfer RNAs (tRNAs) in oxidative stress

The AlkB Homolog 8, tRNA Methyltransferase (ALKBH8), catalyzes mcm^5^U modifications at the wobble position of several tRNAs, including the selenocysteine-specific tRNA (*tRNA*^*Sec*^). Oxidative stress induces the expression of ALKBH8, leading to an increase in translation of several selenocysteine-containing proteins (also known as selenoproteins), including the members of the glutathione peroxidase family (Gpx1, 3, 6 and 4), which are required for ROS detoxification. Mouse embryonic fibroblasts lacking *Alkbh8* exhibited increased intracellular ROS and DNA damage levels (80). Upon treatment with H_2_O_2_, *Alkbh8*-deficient cells displayed elevated lipid peroxidation products compared to wild-type cells (80).

Under oxidative stress, tRNAs undergo retrograde transport from the cytoplasm to the nucleus to suppress protein synthesis and protect the cells from ROS damage (81). Notably, this retrograde transport is selective for certain tRNAs and dependent on the activation of ISR (81, 82). As noted above, studies in yeast demonstrated that oxidative stress could impact tRNA charging levels. In response to peroxide treatment, the charging of tryptophan tRNAs (*tRNA*^*Trp*^) is significantly reduced (66). Ribo-seq of peroxide-treated yeast cells revealed that the reduction in *tRNA*^*Trp*^ charging specifically led to increased codon occupancy and ribosome stalling at UGG codon. The mechanism underlying the change in tRNA charging remains unknown, as total mRNA and protein levels of *WRS1*, the gene encoding tryptophanyl-tRNA synthetase, were unaffected by peroxide treatment.

### mTORC1-dependent activation of GSH synthesis *via* ATF4

Surprisingly, activation of mechanistic target of rapamycin complex 1 (mTORC1) by insulin and growth factors—despite promoting global protein synthesis—also leads to the translational upregulation of ATF4 mRNA through a mechanism that is independent of ATF4’s upstream open reading frames (uORFs) and eIF2α phosphorylation (83, 84). mTORC1 activation exerts a stimulatory effect on a subset of ATF4 target genes that are typically induced by the ISR, including those involved in cellular cystine uptake and GSH synthesis (85). This finding suggests that cooperation between the mTORC1 and ISR pathways is necessary to maintain optimal GSH levels *via* ATF4 during both elevated (mTORC1 activation) and reduced (the ISR activation) global protein synthesis.

### Oxidative stress effects on the translatome

Genome-wide translatome analyses highlight the complexity of translational control in response to oxidative stress. Grant *et al.* assessed global protein synthesis in yeast following oxidative stress induced by H_2_O_2_ (86). General translation is inhibited in response to H_2_O_2_ treatment through both GCN2-dependent and -independent mechanisms. In addition to inducing eIF2α phosphorylation, H_2_O_2_ increases the ribosomal transit time on mRNAs, demonstrating that it suppresses translation at both the initiation and elongation steps. Furthermore, microarray analysis of polysome and monosome fractions revealed that the translation of a subset of mRNAs—including those encoding stress-protective proteins such as cytosolic catalase (*CTT1*) and the atypical 2-Cys peroxiredoxin (*GPX2*)—was increased in response to H_2_O_2_ (86).

Gladyshev and colleagues employed Ribo-seq to assess genome-wide translational control in yeast during exposure to H_2_O_2_ (87). H_2_O_2_ treatment induced several alterations in canonical mRNA translation, including increased ribosome occupancy at short upstream open reading frames (uORFs), N-terminal extension of open reading frames (ORFs), enhanced stop codon read-through, elevated ribosome frameshifting, and increased ribosome occupancy at the start of ORFs, suggesting a potential prolongation of the elongation phase (87). In mammalian cells, treatment with sodium arsenite (NaAsO_2_), which induces ROS production (88) and phosphorylation of eIF2α (89), led to a general inhibition of mRNA translation. However, the translation of a subset of cellular mRNAs, including *ATF4*, *ATF5*, *IFRD1*, *PPP1R15A*, *SLC35A4*, and *C19orf48*, persisted. The 5′UTR region of these mRNAs possesses uORFs, which confer resistance to increased eIF2α phosphorylation (90).

Using Ribo-seq, we showed that eIF2α phosphorylation during mESCs differentiation promotes the translation of mRNAs encoding components of the GSH metabolic pathway including selenoproteins Gpx1 and Gpx3 (91). Interestingly, among these mRNAs, *SLC25A39*, an integral component of the mitochondrial GSH import machinery (92) contains an evolutionarily conserved inhibitory uORF. Additionally, several genes involved in cysteine uptake and GSH synthesis were transcriptionally repressed in eIF2α phosphorylation–deficient cells, including those with confirmed ATF4 binding sites. These findings highlight the complexity of eIF2α phosphorylation in controlling the GSH metabolic pathway at both transcriptional and translational levels.

## Conclusion

Significant progress has been made in understanding the interconnectedness between the evolutionarily conserved ISR pathway and oxidative stress in physiology and disease (93). It is now clear that the ISR pathway plays a crucial role in the cellular response to oxidative stress. The activation of an amino acid biosynthetic program downstream of ISR not only fuel protein synthesis but also provide building blocks for antioxidant response. However, several important questions remain. How do cells sense oxidative stress or ROS to adjust the rate of protein synthesis? As noted above, ribosome stalling in response to oxidative stress mediates the activation of GCN2. Experiments using arsenite have provided strong evidence that HRI is a critical sensor of oxidative stress in various cell types, yet the exact mechanism of HRI activation remains unknown. Other studies have reported that PKR is activated under oxidative stress (94, 95, 96). Do different sources of oxidative stress, such as GSSG and H_2_O_2_, engage a distinct eIF2α kinase or do each of the eIF2α kinases have a different threshold for oxidative stress regardless of its source? Additionally, what is the contribution of translational control downstream of the other eIF2α kinases in the antioxidant response? A deeper biochemical understanding of the interplay between mRNA translational control and oxidative stress is required to address these questions.

## Conflict of interest

The authors declare that they have no conflicts of interest with the contents of this article.
